# Immunohistochemical markers of prognosis in adult granulosa cell tumors of the ovary – a review

**DOI:** 10.1186/s13048-023-01125-1

**Published:** 2023-03-03

**Authors:** Dennis Jung, Katrin Almstedt, Marco J. Battista, Alexander Seeger, Jörg Jäkel, Walburgis Brenner, Annette Hasenburg

**Affiliations:** 1 Department of Gynecology and Obstetrics, University Mainz, Langenbeckstr. 1, Mainz, 55131 Germany; 2 Department of Pathology, University Mainz, Langenbeckstr. 1, Mainz, 55131 Germany

**Keywords:** Adult granulosa cell tumor, Ovary, Prognosis, Immunohistochemistry

## Abstract

**Background:**

Granulosa cell tumors (GCT) are rare malignant ovarian tumors. The two subtypes, adult and juvenile granulosa cell tumors, differ in clinical and molecular characteristics. GCT are low-malignant tumors and are generally associated with favorable prognosis. However, relapses are common even years and decades after diagnosis. Prognostic and predictive factors are difficult to assess in this rare tumor entity. The purpose of this review is to provide a comprehensive overview of the current state of knowledge on prognostic markers of GCT to identify patients with a high risk of recurrence.

**Methods:**

Systematic research for adult ovarian granulosa cell tumors and prognosis revealed *n* = 409 English full text results from 1965 to 2021. Of these articles, *n* = 35 were considered for this review after title and abstract screening and topic-specific matching. A specific search for pathologic markers with prognostic relevance for GCT identified *n* = 19 articles that were added to this review.

**Results:**

FOXL2 mutation and *FOXL2* mRNA were inverse and immunohistochemical (IHC) expression of CD56, GATA-4 and SMAD3 was associated with reduced prognosis. IHC analysis of estrogen receptor, Anti-Mullerian hormone (AMH) and inhibin was not associated with prognosis for GCT. Analyses of mitotic rate, Ki-67, p53, β-catenin and HER2 revealed inconsistent results.

## Introduction

Granulosa cell tumors (GCT) are a rare malignant subtype of ovarian tumors. They comprise about 1–2% of all ovarian neoplasms and 5% of malignant ovarian tumors [1]. There are two subtypes, adult granulosa cell tumors (AGCT), occurring in peri- and postmenopausal women, and juvenile granulosa cell tumors (JGCT), mostly affecting younger patients [1]. AGCT are the more common form (90–95%) compared to JGCT. The leading symptom of GCT is based on the ability to produce estrogens. Potential clinical manifestations are irregular vaginal and postmenopausal bleeding. However, in rare cases, AGCT are accompanied by testosterone and/or androstentione production and result in virilizing symptoms like hirsutism, acne or primary amenorrea in prepubertal patients [2, 3]. Furthermore, nonspecific symptoms like abdominal pain, distension or bloating can occur [4, 5]. Therapy of GCT is based on surgery. The extent of surgery depends on the stage of disease, which is classified analogous to ovarian cancer. Surgical therapy includes at least unilateral salpingo-oophorectomy with simultaneous curettage of the uterus to exclude a concurrent endometrial carcinoma [6]. In postmenopausal women or patients with advanced disease bilateral salpingo-oophorectomy and hysterectomy should be considered [7, 8]. The benefit of adjuvant chemotherapy is being discussed controversially. In advanced stages (FIGO ≥IC) a platinum-based chemotherapy can be conducted [7]. Most tumors (50–80%) are detected in early stages (FIGO Ia) [9] that are accompanied with favorable prognosis with 5-year and 10-year overall survival rates of 97 and 95% [10]. However, recurrence rates are high (10–64%) and relapses can occur years after the initial diagnosis, on average after 48–57 months [11]. For this reason, the German S3-guidline on ovarian cancer recommends life-time follow-up [6]. Due to this unpredictable prognosis of late recurrence, researchers aimed to identify markers to predict prognosis and recurrence. Besides clinical markers (tumor stage, tumor rupture, age and tumor size), pathological markers, that are easy to assess and that provide prognostic information are of clinical interest. In this article, we summarize the current state of knowledge on all published immunohistochemical (IHC) markers and their relevance concerning prognosis with the aim to underline the necessity of further research regarding AGCT.

## Methods

**Fig. 1:**
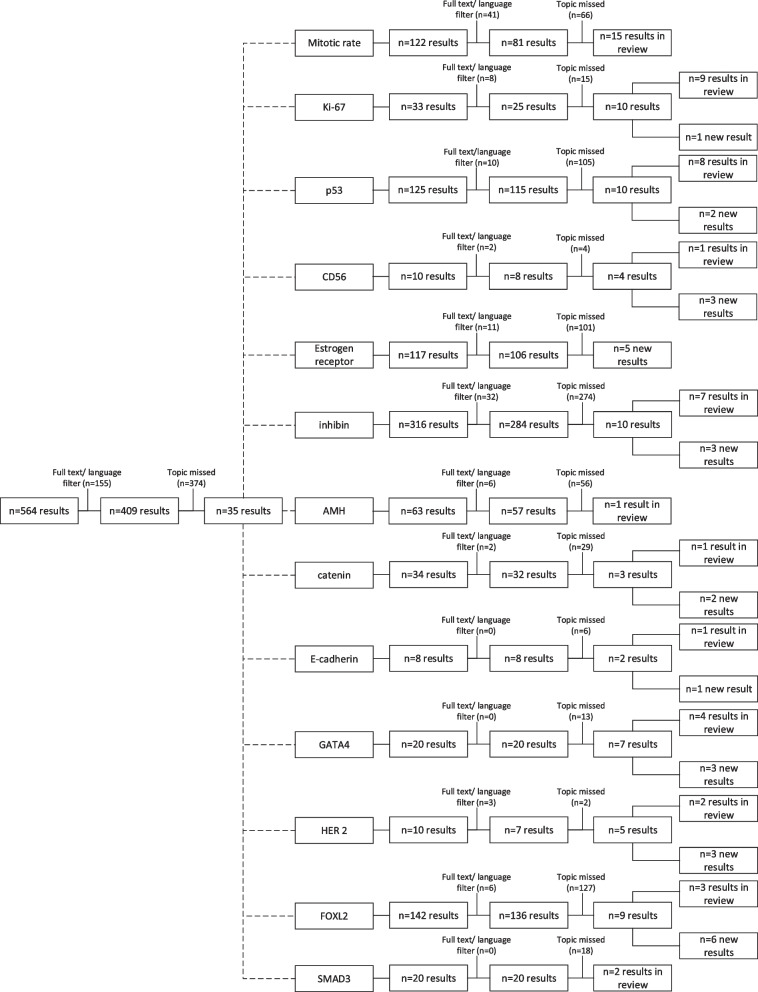
Flowchart of systematic pubmed literature search

Systematic PubMed search for prognosis of ovarian granulosa cell tumors ‘((granulosa cell tumor) AND (ovary OR ovarian)) AND (prognosis OR prognostic)’ added up to 564 results from 1952 to 2021. Filtering only articles with available English full text left 409 results from 1965 to 2021. Three hundred seventy-four articles missed the topic of this review after title and abstract screening, 35 articles referring to pathological markers were considered in this review. The prognostic markers were chosen based on these selected articles. Specific search for the GCT markers ‘((granulosa cell tumor) AND (ovary OR ovarian)) AND (xxx)’; xxx representing ‘mitosis OR mitotic’, ‘Ki-67’, ‘p53’, ‘CD56’, ‘estrogen receptor’, ‘inhibin’, ‘AMH’, ‘catenin’, ‘cadherin’, ‘GATA4’, ‘HER 2’, ‘FOXL2’ and ‘SMAD3’, respectively, confirmed that no articles were missed. Specific search revealed *n* = 1 new article for Ki-67; *n* = 2 for p53, *n* = 3 for CD56, *n* = 3 for inhibin, *n* = 2 for catenin, *n* = 1 for cadherin, *n* = 3 for GATA4, *n* = 3 for HER 2 and *n* = 6 for FOXL2, respectively (Fig. 1). These articles were added to this review. A total of *n* = 54 articles were reviewed for this article.

## Results

### Mitotic rate

The mitotic rate is the number of mitoses traditionally counted in an area of 10 high power fields (HPF). The area with the highest density of mitotic figures is chosen and a light microscope using a 10x ocular and 40x objective magnification is used [12]. Currently, counting a defined area expressed in mm^2^ is advocated by the WHO rather than using HPF due to different microscopes and field diameters [13]. The exact field diameter respectively the area counted was not always stated in the studies evaluated, rendering mitotic count difficult to compare. Despite this, from the 1970s onwards, with a peak in the 1990s, the mitotic rate was numerously evaluated (stated in HPF) as a prognostic marker for AGCT. Some study groups showed a discordant correlation between mitotic count and survival [9, 14–20]. In other studies, significance was not met [21–26]. After all, results are difficult to compare, as different cut-off values and counting areas expressed in HPF were used (> 3/10 high-power field (HPF), > 5/10 HPF and > 10/10 HPF). Furthermore in most studies, stages of disease were not analyzed separately [27] (Table 1). Most AGCT are diagnosed in stage I, which makes a reliable prognosis for this tumor stage most crucial. Studies that did not analyze the stages independently cannot elaborate on the prognosis of early stage AGCT, which is relevant for most of the patients. The heterogeneous results of the different studies excludes the mitotic rate - particularly evaluated in HPF - at the moment as a reliable prognostic factor for AGCT.

**Table 1:** Summary of references analyzing mitotic rate as a prognostic marker

Reference	AGCT/JGCT	Cases (n)	Stage	MI Cut off	Prognostic Significance
Malmström 1994 [14]	Not specified	*n* = 54; assessment of MI in *n* = 42	I (83%); II (11%); III (2%)	≤4 HPF; 5–9 HPF; ≥10 HPF	Survival reduced in subgroup, no *p*-value given
King 1996 [15]	*n* = 38 AGCT; *n* = 2 JGCT	*n* = 40	I (77.5%); II (7.5%); III (15%)	Not specified	Stage (*p* = 0.005) Survival (*p* = 0.006)
Fujimoto 2001 [16]	AGCT	*n* = 27	I (63%); II (15%); III (19%); IV (3%)	4/10 HPF	Survival (*p* < 0.005) Recurrence (*p* < 0.001)
Sehouli 2004 [9]	Not specified	*n* = 65	I (80%); II (7.7%); III (9.2%); IV (3.1%)	5/10 HPF	Survival (*p* < 0.001)
Van Meurs 2014 [17]	AGCT	*n* = 127	I (76%); II-IV (24%)	5/10 HPF	Recurrence (*p* < 0.001)
Thomakos 2016 [18]	AGCT	*n* = 43	I (95%); II (5%)	4/10 HPF	Recurrence (*p* = 0.027)
Sakr 2017 [19]	*n* = 113 AGCT *n* = 12 JGCT	*n* = 125	I-II (95%); III-IV (5%)	4/10 HPF	Recurrence (*p* = 0.021) DFI (*p* = 0.005)
Dridi 2018 [20]	AGCT	*n* = 31; assessment of MI in *n* = 22	I (61%); II (10%); III (19%); IV (10%)	Not specified	Survival (*p* = 0.01)
Costa 1995 [21]	*n* = 49 AGCT *n* = 7 JGCT	*n* = 56	I (84%); II (3%): III (13%)	5/10 HPF	No significance
Lauszus 2001 [22]	Not specified	*n* = 37	I (100%)	Not specified	No significance
Lin 2005 [23]	AGCT	*n* = 36	I (97%); II (3%)	< 1 HPF; 2–3 HPF ≥4 HPF	No significance
Kim 2006 [24]	AGCT	*n* = 35	I (86%); II (3%); III (11%)	Not specified	No significance
Pectasides 2008 [25]	AGCT	*n* = 34	I (59%); II (8%); III (22%); IV (11%)	1–3 HPF; 4–10 HPF; > 10 HPF	No significance
Leuverink 2008 [27]	AGCT	*n* = 38	I (76%); II (11%); III (11%); IV (2%)	Not specified	No significance
Suri 2013 [26]	AGCT	*n* = 104; assessment of MI in *n* = 50	I (95%); II (1%); III (4%)	4/10 HPF	No significance

*AGCT* adult granulosa cell tumor, *JGCT* juvenile granulosa cell tumor, *MI* mitotic index, *HPF* high power field, *HR* hazard ratio, *DFI* disease free interval

### Ki-67

Ki-67 is a nuclear antigen expressed in certain phases of the cell cycle and therefore a marker for evaluating the growth of cell populations. Ki-67 can be detected using a monoclonal antibody [28]. In many tumor entities, the proliferative marker Ki-67 is an important variable for risk classification. However, a considerable inter-laboratory and -observer variability is known. Therefore, it is not surprising that the methodical implementation and the results on Ki-67 also vary in GCT studies. Leuverink et al. reviewed and repeated Ki-67 IHC in *n* = 40 AGCT. To objectify the assessment of this proliferation index they adjusted for inter-observer variation, but could not meet significance for tumor recurrence [27].

One study found significant results concerning Ki-67 and prognosis. Ki-67 was expressed in 12/21 cases, high expression was observed in *n* = 5 cases that correlated with higher tumor stage, but no data on survival or recurrence were presented [29]. Most studies could not find a significant correlation between Ki-67 expression and prognostic data [21, 27, 30–32].

### p53

Historically, p53 has been thought to be an oncogene and mutations of p53 occur frequently and transform p53 into an oncogene (mutant p53, p53m) [33] and accumulates in the nuclei of tumor cells at a detectable amount [34]. Wild type p53 (p53wt) has a short half-life and is not detectable by IHC. Based on this knowledge, studies have analysed p53 IHC expression to correlate the results with prognostic data. In one study, p53 was detected in 13 of 67 GCTs at different amounts, but without prognostic influence [21]. Other study groups reported similar results [15, 30]. Accordingly, in a study by King et al., 75% of cases (*n* = 32) had positive staining for p53m, which did not correlate with stage of disease, recurrence risk or survival [15]. Other study groups found correlation between p53m positivity and prognosis. In the study of Ala-Fossi and colleagues 37% of the tumors (*n* = 30) were positive for p53m. p53 was more common in patients with stage II or higher compared to stage I. Furthermore, the overall survival (OS) of p53-negative tumors was approximately 10 times higher than the median survival of p53-positive patients (267 months vs 21 months, *p* = 0.037) [35]. In this study it needs to be taken into consideration that disease free survival (DFS), which was the primary endpoint in most other studies, has not been analyzed. Secondly, as in most GCT-studies, the number of patients was low, which compromises statistical evidence. Nevertheless, these results were supported by Gebhart et al. showing increased rates of recurrence and decreased progression free survival (PFS) in tumors with overexpressed p53 immunoreactivity. In their study 27/47 tumors (57%) stained positive for p53 [36]. In the study of Staibano et al. 12/30 (40%) GCT (AGCT and JGCT) showed overexpression of p53 which correlated with tumor progression (metastasis and/or death), but this correlation was predominant in the group of JGCT [37]. Contrarily, in the study of Fujimoto et al. p53 was negative in 24/25 cases, suggesting p53 alteration not to be common in AGCT [16], which was supported by other studies [31, 38, 39].

Currently, immunoexpression of p53 is evaluated as follows: staining of 1–80% is regarded as normal (wild type) activity, strong staining of > 80–100% as well as absence of staining in tumor nuclei are regarded as abnormal (mutant). Mutant expression of p53 is considered as a surrogate for *TP53* mutation in ovarian cancer [40]. Roze et al. conducted a whole genome analysis of AGCTs and found a subgroup of AGCT with *TP53* mutation. These tumors were characterized by numerous alterations and increased mitotic activity [41].

To summarize, in older publications, analysis of p53 expression was based on outdated knowledge. Currently, a molecular approach is used to analyze *TP53* mutations, however the prognostic impact is unclear. Also, the previous studies revealed inconsistent results on the influence of p53 as a prognostic marker in AGCT and the relative number of positive tumors throughout the studies varied widely.

### CD56

CD56 (NCAM) is an immunoglobulin participating in organogenesis [42]. Its isoform CD56-140 kDa is involved in the folliculogenesis of the ovary [43]. It is a sensitive diagnostic marker in neuroendocrine tumors, e.g. carcinoid tumors as well as small cell carcinoma of the lung [44], but has also been investigated as a prognostic marker in GCT. Ohishi et al. found all of their *n* = 32 GCT to be positive for CD56, helping to distinguish between different entities of ovarian tumors [45]. Volker et al. examined the staining intensity of CD56pan and its isoforms CD56-140 kDa / -180 kDa in *n* = 30 AGCT (16 primaries and 14 relapses). They were able to show an increased staining intensity of the high molecular CD56 isoforms in relapses and relapsing primaries compared with CD56pan in unrelapsed primaries. They concluded this molecular isoform to be a possible sign for a more aggressive behavior of the tumors [46]. In the study of Sakr et al. high expression of CD56pan was significantly associated with higher recurrence rate and decreased disease free interval (DFI) (156.8 months vs 453.9 months, *p* = 0.001) [19].

### Estrogen receptor

Studies have shown that estrogen plays an important role in carcinogenesis of ovarian neoplasms [47]. Two types of estrogen receptors (ER) are expressed in the ovaries, ER-⍺ and ER-β. In normal ovarian tissues, both are expressed in comparable levels. However, in ovarian carcinomas, this ratio seems to shift towards ER-⍺, as in these samples lower levels of ER-β are detected [48, 49]. In a study of *n* = 30 GCT (19 AGCT and 11 JGCT), Staibano et al. examined the expression of ER-β. In five cases, expression of ER-β was scored negative, eight cases showed low expression, in 10 cases medium expression was found and seven cases revealed high expression of ER-β. These results were compared with follow-up data. Loss of ER-β was significantly associated with worse prognosis [37]. Contradictingly, Puechl et al. examined ER (no differentiation between subtypes ER-⍺ and ER-β) and progesterone receptor (PR) expression in *n* = 149 AGCT of a multicenter study. They did not find a correlation between the expression of ER and prognosis. However, PR expression showed to be a predictor of recurrence free survival (RFS) and OS. In their study, a high PR expression score was significantly associated with worse RFS and OS [50]. Balan et al. identified nine of 21 cases with positive staining for ER-⍺ with mixed staining intensity. They concluded that their results showed no significant correlation with prognosis [29]. Another type of receptor, the G-protein coupled estrogen receptor (GPER), had already been analyzed in ovarian carcinoma by Heublein et al. [51]. The same study group analyzed its impact on prognosis in GCT. They found a positive staining of GPER in 53.8% (14/26) and high intensity staining in 26.9% (7/26). The expression of GPER was related to reduced OS. Primary-diagnosed patients with high intensity of GPER staining had significantly reduced OS [52].

### Inhibin

Inhibin is a glycoprotein hormone that is produced in granulosa cells of the ovary. It is a heterodimer consisting of α and β dimers. The β dimer is divided in two subunits βA and βB, differentiating between inhibin-A and inhibin-B. It is responsible for suppressing the secretion of follicle-stimulating hormone (FSH) by the pituitary gland via a feedback system [53]. As shown by Gurusinghe et al. it is not only measurable in serum, but also detectable by IHC in ovarian (tumor) tissue [54]. In normal ovaries, the expression of inhibin can be seen in the cytoplasm of granulosa cells, theca interna cells, Sertoli cells and Leydig cells [55]. In malignant ovarian neoplasms it is reported that inhibin is highly expressed in sex cord stromal tumors, i.e. GCT and Sertoli-Leydig cell tumors, whereas other ovarian carcinoma subtypes are mostly negative. Therefore the inhibin expression helps to distinguish sex cord stromal tumors from other ovarian malignant neoplasms [56, 57]. Gebhart et al. were able to detect inhibin-α in 42 of 47 GCT (89%); 57% were stained strongly, 21% moderately and 10% weakly. Of all cases, most tumors (83%) were stage I. For this reason, stages II and III were grouped for statistical analysis. The percentage of tumor cells that stained positively for inhibin was defined as staining reactivity. Decreased staining reactivity and intensity for inhibin were associated with advanced stages of disease. However, the results did not correlate with survival (PFS) [36]. These results are in accordance with Balan et al. who found 14 of 21 GCT (66.66%) positive for inhibin-α. According to the results of this working group, the expression of inhibin appeared to inversely correlate with tumor aggressiveness [29]. This was supported by the statements of Matzuk et al. who suggested inhibin to be a tumor suppressor gene, as they were able to show an increased development of gonadal tumors in inhibin-deficient mice [58]. Contrarily, Sakr et al. found out that increased expression of inhibin-α was associated with increased disease recurrence [19]. However, another study was not able to correlate IHC expression of inhibin-α with prognosis. In the study of Anttonen et al. all tumors (*n* = 80) except for three stained positive for inhibin-α, but data failed to correlate with recurrence risk, stage or prognosis [59].

### Anti-Mullerian hormone (AMH)

Anti-Mullerian hormone (AMH), also known as Mullerian inhibiting substance (MIS), is a growth factor produced in the gonads and is responsible for folliculogenesis and sexual differentiation [60]. It was identified as a serum marker for GCT; diagnostic is also verified for this tumor entity by IHC [61]. Literature search revealed one study concerning IHC of AMH and prognosis. In this study, reduced AMH expression correlated only with larger tumor size, but not with prognosis (a. e. recurrence risk) [59]. In summary, the prognostic value of AMH-IHC remains unclear; however, it is a well-established serum marker for therapy monitoring and patients follow-up [62].

### E-cadherin, ß-catenin

E-cadherin is a transmembrane protein responsible for cell-cell adhesion. Through a cytoplasmic binding site, the catenin binding domain (CBD) β-catenin controls and modulates E-cadherin function [63]. When activated by wnt-signaling, β-catenin is responsible for target gene expression after it translocates into the nucleus [64]. It is suggested that downregulation of E-cadherin promotes tumor progression in most solid tumor types [65]. Boerboom et al. found that misregulation of β-catenin via the wnt signaling pathway results in GCT transformation [66]. The working group detected mutant β-catenin in the nuclei of human (*n* = 1 of 6) and equine (*n* = 14 of 18) GCT, but not in normal ovarian tissue samples. These results were refuted by Ohishi et al. who did not find nuclear expression of β-catenin (*n* = 0 of 30 AGCT), which contradicts the hypothesis that nuclear β-catenin supports tumor progression in AGCT. Rather they found nuclear expression of E-cadherin (*n* = 27 of 30 AGCT), which is usually located at the cell membrane. However, nuclear E-cadherin expression was not associated with prognosis [67]. Stewart et al. did also analyze E-cadherin and β-catenin expression in AGCT, FIGO stage I (*n* = 62), and its influence on prognosis. They detected β-catenin expression in all AGCT samples and E-cadherin expression in 85%. E-cadherin staining was mostly restricted to sex cord-like components of the tumors and was in general weaker in extent and intensity than β-catenin. In cells with strong E-cadherin expression, staining was prevailing in the membrane, whereas cells with weaker staining showed more cytoplasmic staining activity. Consistent to Ohishi et al., Stewart et al. did not find nuclear β-catenin expression. In correlation with patients clinical outcome, they proved that less extensive β-catenin staining was associated with a higher rate of AGCT recurrence and shorter DFS compared to a more extensive staining. No clinical correlation was found to cytoplasmic β-catenin staining intensity as well as both, E-cadherin extent and intensity [68].

### GATA-4

GATA-4 is a zinc-finger transcription factor that is responsible for various genes in the steroidogenesis and normal granulosa cell function [69–71]. It has also been shown that GATA-4 regulates cell apoptosis in GCT by escaping TRAIL (Tumor necrosis factor-related apoptosis-inducing ligand)-induced apoptosis and by activating apoptosis inhibitor BCL-2 [72–74]. In the study of Anttonen et al. high GATA-4 expression was seen in 44% of GCT tumors compared to granulosa cells of normal ovarian tissue samples. Increased GATA-4 expression was associated with advanced tumor stages and risk of tumor recurrence. 14 of 80 patients had disease recurrence of which all had positive GATA-4 expression in the primary tumors (*n* = 11 with high expression, *n* = 3 with intermediate expression). In the same tumor samples, opposite results were shown for GATA-6. Expression of GATA-6 was shown to be reduced in GCT. Consistently with AMH, reduced GATA-6 expression correlated with larger tumor size, but not with prognosis [59]. Likewise Färkkilä et al. found an association between expression level of GATA-4 and tumor stage (Ib-III) and prognosis, respectively. High GATA-4 was associated with a reduced DFS, independently of tumor stage [75]. In contrast, Sakr et al. could not find any prognostic significance of GATA-4 [19].

### HER2

Human epidermal growth factor receptor (HER2) is a member of the epidermal growth factor receptor (EGFR) family [76]. It is a well-established diagnostic and therapeutic target in breast cancer [77] and gastric cancer [78]. HER2 was investigated as a potential target and prognostic factor in GCT. Leibl et al. analyzed the expression of EGFRs: HER1/EGFR1, HER2, HER3 and HER4 in GCT immunohistochemically. They were able to show positive staining of HER1/EGFR1 (65.0%), HER3 (45.0%) and HER4 (57.5%). HER2 was not expressed in any of the *n* = 40 GCT tumor samples [79]. These results were supported by two further working groups. Higgins et al. examined *n* = 31 cases of AGCT and found positive staining of HER1/EGFR1 in 23 cases (74.2%), but negative staining results for HER2 in all samples [80]. Menczer et al. did not detect any HER2 expression in 13 analyzed GCT) either [81]. In contrast, three other studies reported positive staining of HER2 in GCT [15, 82, 83]. Färkkilä et al. also analyzed HER2 expression in AGCT and found positive staining in 98% of the tumors. Expression of HER2 correlated with tumor stage and tumor recurrence. Furthermore, a co-expression of HER2 and GATA-4 was observed. HER2 and GATA-4 showed a negative prognostic effect (DFS), which was enhanced when expressed simultaneously [75]. This was also supported by Sakr et al. [19].

### FOXL2

FOXL2 is a member of the forkhead transcription factors and is involved in embryogenesis and ovarian differentiation as well as granulosa cell differentiation and follicle development [84, 85]. In 2009, a somatic missense point mutation (402C > G) was detected in 97% of AGCT and identified as a promotor of granulosa cell tumor pathogenesis [86, 87]. The mutant *FOXL2* results in an alteration of its pro-apoptotic function [88], and the induction of anti-proliferative factors like follistatin is inhibited [71, 89, 90]. Autosomal dominant mutation of *FOXL2* gene is also associated with blepharophimosis-ptosis-epicanthus-inversus syndrome (BPES) which manifests in two forms, BPES type II resulting in isolated craniofacial abnormalities and BPES type I additionally being accompanied by premature ovarian failure [91, 92]. D’Angelo et al. investigated the influence of *FOXL2* on prognosis. They showed that *FOXL2* mutation (402C > G), which was detected in 70% of cases, correlated with a poor prognosis (DFS). DFS was also reduced in patients with increased *FOXL2* mRNA expression. In IHC staining, reactivity for FOXL2 was higher in tumor samples expressing mutant *FOXL2*, but was not associated with prognosis (DFS or OS) in AGCT. Contrarily, in JGCT, where *FOXL2* mutation is rare, strong FOXL2 immunoreactivity correlated with decreased DFS and OS [93]. Kraus et al. found *FOXL2* (402C > G) mutation in 38 of 40 AGCT. Three of the recurrent tumors exhibited homozygous genotype. The authors concluded that *FOXL2* homozygous genotype is more likely to relapse than heterozygous genotype [94]. In a sample of *n* = 26 patients with AGCT, Rosario et al. were also not able to find significant correlation between *FOXL2* mutation and tumor size or prognosis [95].

### SMAD3 (mothers against decapentaplegic homolog 3)

SMAD3 is a mediator of transforming growth factor beta (TGFβ)-function. It is responsible for cell viability in AGCT [96]. SMAD3 works as cooperator of GATA-4 in the TGFβ pathway being responsible for inhibin-α activation [97]. Synergistically with GATA-4 it activates the cyclin D2 (CCND2) promoter, a key factor for proliferation and survival in granulosa cell tumors [71]. In a study with *n* = 88 primary GCT cases, Sakr et al. showed that increased expression of SMAD3 was significantly associated with increased recurrence and a shorter DFI (220.6 vs. 441.5 months, *p* = 0.001). SMAD3 was also revealed as a predictor of recurrence in GCT (OR = 14.2, *p* = 0.001) [19].

## Discussion

This review gives an overview about the multiple pathways and molecular factors and their prognostic role in AGCT using IHC staining. Numerous studies pointed out different factors and mutations that were associated with proliferation or tumorigenesis of AGCT and analyzed their IHC expression. The studies were confronted with various challenges. The two different entities, AGCT and JGCT, vary widely in characteristics. AGCT, the more common form of GCT, can occur at all ages with a peak in perimenopausal women, whereas JGCTs commonly occur before the age of 30. Clinical behavior also differs between these tumor types. Both tumors are associated with a good prognosis, but relapses are common. AGCT tend to recur late, even later than 10 to 20 years after diagnosis, JGCT generally within a few years [98, 99]. As stated before, therefore guidelines recommend life-long follow-up, which for most women is associated with concerns about disease recurrence [6]. For this reason, it is necessary to identify patients at high or low risk of recurrence in order to provide individualized follow-up programs. Knowledge of molecular pathways associated with severe disease progression may also lead to new (targeted) therapeutic opportunities.

The greatest challenge in the study of GCT is its low incidence. As AGCTs, and even more JGCT, are rare tumors, statistically significant results are difficult to obtain due to the low number of cases. Differentiation between the individual tumor stages is often not possible. Many of the reviewed studies in this article did not differentiate between AGCT and JGCT. Regarding the molecular and prognostic differences of these subtypes, results for prognostic IHC markers are difficult to obtain. No significant correlation between pathological markers and prognosis was found concerning ER- and inhibin-expression. In regard to the mitotic rate, Ki-67, p53, β-catenin, and HER2, the results of the individual studies were contradictory (Table 2), probably also due to interpretation problems, especially regarding p53 IHC as mutation-positive in older studies. In particular, HER2 is known as a predictive and prognostic factor. A variety of potent targeted therapies against the HER2 receptor already exist. Further investigation of this receptor in GCT is therefore of oncological importance for individual therapeutic concepts. Ki-67 IHC revealed conflicting data. With the exception of one study, a correlation between IHC expression of Ki-67 and prognosis was not found. However, a considerable inter-observer variation is known and has not been acknowledged in most studies. The significance of Ki-67 as a prognostic marker has been classified differently in different studies. In breast cancer, Ki-67 is a well-established prognostic marker. A cut-off value has been defined to differentiate between luminal A and luminal B tumor types [100]. In none of the reviewed studies, cut-off values were determined for GCT. Expression was only distinguished between high and low; e.g. Mayr et al. detected a Ki-67 index < 5% in half of their cases (*n* = 10) and an index between 5 and 25% in 45% (*n* = 9) [30]. Further studies with standardized methodology and elimination of Ki-67 variabilities may help to define the prognostic value of this proliferation marker.

**Table 2:** Summary of the reviewed markers (alphabetical order) and their prognostic significance

Marker	Number of references reviewed in article	Conclusion	Notes
Anti-Mullerian hormone (AMH)	*n* = 1	Prognostic significance unclear	- Correlation of AMH expression with larger tumor size, but not with prognostic data
**CD56**	*n* = 3	**Prognostic significance**	- High IHC expression associated with increased recurrence and decreased DFI
E-cadherin, β-Catenin	*n* = 3	Prognostic significance unclear	- conflicting data on expression and prognostic validity
Estrogen	*n* = 4	No prognostic significance	- Studies with conflicting results
**FOXL2**	*n* = 3	**Prognostic significance**	- FOXL2 expression is associated with decreased DFS and OS in JGCT - FOXL2 mutation and FOXL2 mRNA are associated with reduced DFS in AGCT
**GATA-4**	*n* = 3	**Prognostic significance**	- High expression of GATA-4 is associated with reduced DFS, higher tumor stage and recurrence
HER2	*n* = 8	Prognostic significance unclear	- conflicting data on IHC expression of HER2
Inhibin	*n* = 5	No prognostic significance	- Studies with significant and insignificant results
Ki-67	*n* = 6	Prognostic significance unclear	- Studies with significant and insignificant results - variations, e.g. inter-observer variation not considered in most studies
Mitotic rate	*n* = 15	Prognostic significance unclear	- Studies with significant and insignificant results - different cut-off values - different microscopes and field diameters - currently counted in mm^2^
p53	*n* = 10	Prognostic significance unclear	- conflicting results of p53 IHC expression - interpretation problems regarding p53 IHC as mutation-positive
**SMAD3**	*n* = 1	**Prognostic significance**	- High expression of SMAD3 is associated with increased recurrence and shorter DFI

*IHC* immunohistochemistry, *DFI* disease free interval, *DFS* disease free survival, *OS* overall survival, *AGCT* adult granulosa cell tumor, *JGCT* juvenile granulosa cell tumor

The prognostic relevance of AMH-IHC remains unclear as in only one study, AMH expression correlated with larger tumor size, but not with prognosis. FOXL2-IHC correlated with decreased DFS and OS in JGCT and *FOXL2* mutation and increased *FOXL2* mRNA were associated with reduced DFS in AGCT.

In recent studies whole genome sequencing was performed and yielded new aspects, such as *TP53* and *FOXL2* mutation. In further studies a new approach including both, immunohistochemical and molecular data might improve assessment of prognosis [101].

## Conclusion

Of all examined markers, this review only revealed a prognostic value for worse outcome of CD56, GATA-4 and SMAD3. To gain more knowledge about this rare tumor entity and its prognosis, large multi-center studies with higher case numbers and clear distinction between AGCT and JGCT are needed. The implementation of national and international tumor registries represents a great opportunity for further evaluation.
